# Physical activity, anxiety, depression, and coping in Turkish men and women during the first wave of COVID-19

**DOI:** 10.1590/0102-311XEN057123

**Published:** 2024-06-14

**Authors:** Bijen Filiz, Funda Coşkun Özyol, Bengü Güven, Ezel Nur Korur, Yılmaz Yüksel, Cavit Işık Yavuz, Kele Ding, Jingzhen Yang, J. Larry Durstine, Ming-kai Chin, Gıyasettin Demirhan

**Affiliations:** 1 Afyon Kocatepe University, Afyonkarahisar, Turkey.; 2 School of Physical Education and Sports, Van Yüzüncü Yıl University, Van, Turkey.; 3 Exercise and Sport Sciences, Başkent University, Ankara, Turkey.; 4 School of Physical Education and Sports, Ordu University, Ordu, Turkey.; 5 Hasan Doğan School of Physical Education and Sports, Karabük University, Karabük, Turkey.; 6 Faculty of Medicine, Hacettepe University, Ankara, Turkey.; 7 School of Health Sciences, Kent State University, Kent, U.S.A.; 8 College of Public Health, Ohio State University, Columbus, U.S.A.; 9 Exercise Science Arnold School of Public Health, University of South Carolina, Columbia, U.S.A.; 10 Foundation for Global Community Health, Las Vegas, U.S.A.; 11 Faculty of Sport Sciences, Hacettepe University, Ankara, Turkey.

**Keywords:** COVID-19, Physical Activity, Depression, Hope, COVID-19, Atividade Física, Depressão, Esperança, COVID-19, Actividad Física, Depresión, Esperanza

## Abstract

A need exists to better understand the relationships between COVID-19, coping behaviors, physical activity and stress, and COVID-19’s impact on way of life. A cross-sectional study design was used to examine adult physical activity, hope, depression, anxiety, and coping status by gender during the COVID-19 pandemic, and to determine the impact of these variables on the coping process. The study also examined the effect of gender on the relation between physical activity and dependent variables. A global survey instrument was used in this study, including 1,400 Turkish adults. This study identified significant gender-based differences regarding physical activity, hope, depression, anxiety, and coping status of adults, although no significant gender-based difference was found regarding hope scores. Furthermore, physical activity directly influenced coping (β = 0.10), hope (β = 0.12), and anxiety (β = -0.08). Hope directly and positively influenced coping (β = 0.45) and directly and negatively influenced anxiety (β = -0.25) and depression (β = -0.28). Moreover, gender did not directly affect physical activity, but it was associated with decreased coping and increased depression and anxiety. Finally, gender had no effect on the relation between physical activity and hope, coping, depression, and anxiety (p > 0.01). These outcomes support the critical importance of physical activity and hope when coping with COVID-19 regardless of gender.

## Resumo

Existe uma necessidade de entender melhor as relações entre a COVID-19, comportamentos de enfrentamento, atividade física e estresse e o impacto da COVID-19 no modo de vida. Um desenho de estudo transversal foi usado para examinar a atividade física adulta, esperança, depressão, ansiedade e estado de enfrentamento por gênero durante a COVID-19 e para determinar os efeitos de atividade física, esperança, depressão, e ansiedade no enfrentamento da COVID-19. Finalmente, examinou-se o efeito do gênero na relação entre atividade física e variáveis dependentes. Um instrumento de pesquisa global foi utilizado neste estudo, no qual um total de 1.400 adultos turcos participaram. Os resultados desta investigação demonstram que existem diferenças significativas em atividade física, esperança, depressão, ansiedade e estado de enfrentamento de adultos por sexo. Não houve diferença significativa entre os sexos para os escores de esperança. Além disso, a atividade física influenciou diretamente o enfrentamento (β = 0,10), a esperança (β = 0,12) e a ansiedade (β = -0,08). A esperança influenciou direta e positivamente o enfrentamento (β = 0,45) e influenciou direta e negativamente a ansiedade (β = -0,25) e a depressão (β = -0,28). Além disso, o gênero não afetou diretamente a atividade física, mas o gênero foi associado à diminuição do enfrentamento e ao aumento da depressão e ansiedade. Finalmente, o gênero não teve efeito sobre a relação entre atividade física e esperança, enfrentamento, depressão, ansiedade (p > 0,01). Estes resultados apoiam a importância crítica da atividade física e da esperança ao lidar com COVID-19 sem efeitos de gênero.

## Resumen

Existe la necesidad de comprender mejor las relaciones entre COVID-19, los comportamientos de afrontamiento, la actividad física y el estrés, y el impacto de COVID-19 en la forma de vida. Se utilizó un diseño de estudio transversal para examinar la actividad física del adulto, la esperanza, la depresión, la ansiedad y el estado de afrontamiento por género durante COVID-19 y para determinar los efectos de la actividad física, la esperanza, la depresión, y ansiedad en el afrontamiento de COVID-19. Finalmente, se examinó el efecto del género en la relación entre la actividad física y las variables dependientes. En este estudio se utilizó un instrumento de investigación global, en el que participaron un total de 1.400 adultos turcos. Los resultados de esta investigación demuestran que existen diferencias significativas en la actividad física, la esperanza, la depresión, la ansiedad y el estado de afrontamiento de los adultos por sexo. No hubo diferencias significativas entre los sexos para las puntuaciones de esperanza. Además, la actividad física influyó directamente en el afrontamiento (β = 0,10), la esperanza (β = 0,12) y la ansiedad (β = -0,08). La esperanza influyó directa y positivamente en el afrontamiento (β = 0,45) e influyó directa y negativamente en la ansiedad (β = -0,25) y la depresión (β = -0,28). Además, el género no afectó directamente a la actividad física, pero el género se asoció con una disminución del afrontamiento y a un aumento de la depresión y la ansiedad. Finalmente, el género no tuvo ningún efecto sobre la relación entre la actividad física y la esperanza, el afrontamiento, la depresión, la ansiedad (p > 0,01). Estos resultados respaldan la importancia crítica de la actividad física y la esperanza cuando se trata de COVID-19 sin efectos de género.

## Introduction

Self-isolation and social distancing became the new normal during the SARS-CoV-2 or coronavirus disease 2019 (COVID-19) pandemic as people learned to cope with daily realities associated with loneliness and psychological distress ^1^. The World Health Organization (WHO) reports that restrictions imposed during the COVID-19 pandemic negatively affected one’s physical and mental health, leading to increased fear, anxiety, and stress. WHO ^2^ conceptualizes mental health as a “*state of well-being in which the individual realizes their own abilities, can cope with the normal stresses of life, can work productively and fruitfully, and is able to make a contribution to their community*”. Salari et al. ^3^ analyzed 17 studies concerning COVID-19 and the prevalence of stress, anxiety, and depression, and concluded that COVID-19 resulted in numerous psychological disorders. Lakhan et al. ^4^ examined 16 studies evaluating 113,285 subjects from China, India, Spain, Italy, and Iran reporting prevalences of 20%, 35%, and 53% for depression, anxiety, and stress, respectively. Özdin et al. ^5^ found that 23.6% of adults from a Turkish community experienced depression and 45.1% of them experienced anxiety during the COVID-19 pandemic. However, Muñoz-Violant et al. ^6^ revealed that stress causing anxious symptomatology rather than a real risk of being sick, has a great effect on people being afraid of disease. These studies provide important information regarding mental well-being importance, especially during pandemics.

Previous information suggests that depression, anxiety, and stress are clearly associated with COVID-19 with similar impacts reported in other countries ^3^ ^,^ ^4^. Risk factors associated with depression and anxiety include gender, various disease risk, presence of chronic or psychiatric disorders, frequent exposure to social media, and news about COVID-19 ^3^.

The WHO ^7^ has made the following recommendations for societal mental health protection: healthy diet, regular and adequate sleep, excessive alcohol consumption avoidance, quit smoking, and daily physical activity. In addition to numerous physiologic benefits to human health ^8^, physical activity reduces anxiety and depression levels, positively impacting mental health ^9^ ^,^ ^10^ ^,^ ^11^.

Hope, a feeling of expectation and desire for a certain event, is another factor contributing to improved mental health during the pandemic. An increase in the feeling of hope enhances the ability to cope with traumatic events and is a protective factor against depression ^12^. In this context, COVID-19 provides an adverse physical environment revealing individuals vulnerable to diseases, while emphasizing hope to cope and survive dramatic difficulties. Hope is essential to maintain an optimistic state of mind when coping with chronic health conditions. Hope, defined as an internal psychologic resource and cognitive feature against anxiety and stress-related disorders ^12^, is a coping mechanism that increases well-being during global health crisis ^13^. Evidence supports that higher levels of hope are associated with lower anxiety ^14^ and stress ^15^. Better coping is associated with reduced depression ^16^. Current evidence concerning the protective role of coping and hope against depression is inadequate, thus limiting further inferences. Therefore, further research is needed to better understand the effects of traumatic health challenges such as COVID-19 and depression. One research path is to explore the potential mediating roles of coping and hope. Considering existing data, it is necessary to better understand the relations between COVID-19, physical activity, stress, and coping behaviors including hope.

For this reason, providing added information to improve public health and mental well-being contributes to the development of appropriate recommendations for long-term lifestyle changes. This study had three main objectives: (1) to examine physical activity, hope, depression, anxiety, and coping levels of adults by gender during the COVID-19 pandemic, (2) to determine the effects of physical activity, hope, depression, and anxiety on coping with COVID-19, and (3) to determine gender effect on the relation between physical activity and hope, coping, depression, and anxiety.

## Methodology

### Study design

This study is part of the global comparison project between 11 countries ^15^ ^,^ ^17^ examining leisure physical activity during the COVID-19 pandemic. Data collected from the cross-sectional online survey with Turkish adults aged ^18^ years or older were included and analyzed. Participant recruitment approaches, data collection methods, and study instruments used in this study matched the global comparison project design ^15^ ^,^ ^17^.

### Participants and procedure

In total, 1,400 adult volunteers met the inclusion criteria, which included being aged over 18 and able to complete a survey. Participants were asked their age, and were able to continue completing the survey if they met age criteria. Then they provided consent and were informed that they could interrupt study participation or skip a question at any time. The survey took approximately 20 minutes to completion and was shared via emails and social networks (Facebook, WhatsApp, LinkedIn, Twitter, and Instagram), employing snowball sampling. Participants were asked to forward the invitation link to their friends and relatives via social networks. Data collection took 60 days, from July 1, 2020 to August 31, 2020. A total of 778 study participants (55.6%) were women and 622 (44.4%) were men.

### Data collection tools

A global survey instrument developed by Ding et al. ^17^ was used with Dr. Ding’s consent to use and to adapt the instrument by adding questions. The survey was translated into Turkish by an English language expert, checked for accuracy, decrypted, and submitted to three other experts for content validity ^18^. To assess the survey validity, the agreement level of experts was calculated reaching 88%. Then, the survey was revised according to the experts’ suggestions. A preliminary survey was administered to 100 adults to add feedback. The final survey included 73 questions, along with skip-pattern questions, and it was prepared using the open-source online survey tool Limesurvey. The instruments and variables used in this study are: *International Physical Activity Questionnaire* short form, *Herth Hope Index*, Brief Resilient Coping Scale, and emotional distress-anxiety/depression-short form, 

####  International Physical Activity Questionnaire short form 

Participants were asked to indicate the frequency and duration of leisure time physical activities during COVID-19 lockdowns using the *International Physical Activity Questionnaire* (IPAQ). Frequency is measured in days per week and length as time per day, based on three intensity levels: vigorous-intensity activities, moderate-intensity activities, and walking. IPAQ was developed by Craig et al. ^19^ to determine physical activity level. The IPAQ was validated among individuals aged 15 to 69 years with established concurrent and criterion validity in several languages, resulting in a test-retest reliability of 0.80 or higher ^19^ ^,^ ^20^. The IPAQ was adapted to the Turkish language by Sağlam et al. ^21^ with reliability coefficients ranging 0.64-0.78. The IPAQ short version consisted of seven items and included information for the last seven days. Total score was calculated by multiplying the minutes (min), days, and metabolic equivalent of task (MET) values of the activities, and is expressed as “MET-min/week”. Vigorous physical activities were multiplied by eight METs; moderate-intensity physical activities multiplied by four METs; and walking was multiplied by 3.3 METs. A total physical activity score (MET-min/week) below 600 indicated low (1) physical activity, between 600-3,000 indicated moderate; (2) physical activity, and above 3,000 indicated high; (3) physical activity level. In our data analysis, low, moderate, and high variables were used for physical activity level. A 0.78 Cronbach’s α was achieved for this scale.

####  Herth Hope Index 

The *Herth Hope Index* was developed by Herth ^22^ (Cronbach’s α of 0.97) and adapted to the Turkish language by Aslan et al. ^23^ (Cronbach’s α of 0.75), totaling 12 items. With this index, participants were asked about their desires and beliefs for a positive future during the lockdown. As an example, items “I can see possibilities in the midst of difficulties” and “I believe that each day has potential” are presented. The scale has a 4-point Likert design: “strongly disagree, disagree, agree, and strongly agree”. Total scores for hope (minimun = 12, maximum = 48) were calculated as a continous variable, with higher scores indicating a higher level of hope. After calculation, a 0.64 Cronbach’s α was achieved for this scale. This value may have been lower in the adaptation study on Turkish population due to cultural differences (0.75 and 0.64).

#### Brief resilient coping scale

The scale developed by Sinclair & Wallston ^24^ (Cronbach’s α of 0.70) consisted of four items, e.g., “I look for creative ways to alter difficult situations” and “I actively look for ways to replace the losses I encounter in life” can be given. The scale consists of a symmetrical 5-point Likert design “never, rarely, sometimes, often, and always”. A back translation was used to adapt the scale to Turkish, and the Turkish form was obtained from a bilingual education specialist’s evaluation. For this scale, the following values were obtained: Cronbach’s α of 0.76 and fit indices of χ^2^/standard deviation (SD) = 3.27, root mean square error of aproximation (RMSA) = 0.040, standardized root mean square residuals (SRMR) = 0.013, normed fit index (NFI) = 1.00, nonnormed fit index (NNFI) = 0.99, and comparative fit index (CFI) = 1.00 (χ^2^ = 6.55, df = 2, p = 0.03). With this index, participants were asked to respond to questions based on how they felt during the lockdown. The total score of the index (minimum = 4, maximum = 20) was calculated, with higher scores indicating higher levels of resilient coping.

#### Emotional distress-anxiety/depression-short form

Pilkonis et al. ^25^ report on item banks development and calibration for depression and anxiety as part of the Patient-Reported Outcomes Measurement Information System (PROMIS). The instrument consisted of two parts: anxiety (Cronbach’s α of 0.93) and depression (Cronbach’s α of 0.95). Four questions were included to each. The questions in the instrument ranking were created to measure participant anxiety and depression in the last seven days. Participants were asked to respond to the index by considering their feelings and thoughts during the lockdown. For example, items for anxiety “I felt fearful” and for depression “I felt depressed” can be given. The scale consists of a symmetrical 5-point Likert design “never, rarely, sometimes, often, and always” (minimum = 4, maximum = 20) with higher scores representing greater anxiety or depression symptoms. Back-translation was used to adapt the scale to Turkish, and the Turkish form was obtained as a result of a bilingual education specialist’s evaluation. For data analysis, the original raw total scores were converted to t scores ^25^. Cronbach’s α of 0.90 for anxiety and 0.87 for depression were calculated; and they fit two-factor confirmatory factor analysis (CFA) model for two dimensions, and the fit indices supported their coherence, χ^2^/SD = 5.68, RMSA = 0.058, SRMR = 0.017, NFI = 0.99, NNFI = 0.99, and CFI = 0.99 (χ^2^ = 90.68, df = 16, p = 0.00) for this scale.

### Data analysis

Data analysis began with standard procedures for data cleaning and screening ^26^. After excluding 44 participants who did not complete the survey, 1,400 surveys were retained for further analysis. Independent t-test was used to determine the differences between physical activity, hope, depression, anxiety, and coping levels by gender. In addition, structural equation modeling was used to determine the effects of physical activity, hope, depression, and anxiety on coping levels during the COVID-19 pandemic. Finally, mediation analysis was used to determine gender effect to physical activity and hope, coping, depression, anxiety relation (intervening-mediator variable = gender; independent variable = physical activity; dependent variables = hope, coping, depression, anxiety). The significance level was set at p = 0.01 for physical activity levels and 0.05 for other analyses. The IBM Amos version 24.0 (https://www.ibm.com/support/pages/downloading-ibm-spss-amos-24), SPSS version 23.0 (https://www.ibm.com/), and SPSS Process Macro Model 1 were used to analyze the study data.

### Ethical aspects

The study was conducted in accordance with the *Declaration of Helsinki*. In this context, the study proposal was approved by the Turkish Ministry of Health and Hacettepe University Ethics Committee (Ministry of Health approval n. 2020-05-23T13_42_55; Ethics Committee approval n. 22.06.2020/82416169-050.06/00001124935). Informed consent was obtained from all participants.

## Results

Table 1 shows significant differences in physical activity levels (t = 5.481, p < 0.05), depression (t = -9.305, p < 0.05), anxiety (t = -11.600, p < 0.05), and coping (t = 4.127, p < 0.05) scores were observed by gender. No significant difference was found by gender (t = 1.624, p > 0.05) for hope scores.

**Table 1:** Results of physical activity, hope, depression, anxiety, and coping levels of adults by gender.

Gender	n	Physical activity [mean (SD)]	Hope [mean (SD)]	Depression [mean (SD)]	Anxiety [mean (SD)]	Coping [mean (SD)]
Men	622	1.88 (0.86)	3.45 (0.48)	47.30 (9.12)	46.68 (8.92)	15.70 (2.70)
Women	778	1.64 (0.80)	3.40 (0.43)	52.16 (10.15)	52.65 (10.03)	15.13 (2.45)
t test	-	5.481	1.624	-9.305	-11.600	4.127
p-value	-	0.00 *	0.10	0.00 *	0.00 *	0.00 *

SD: standard deviation.

* p < 0.05.

**Figure 1:**
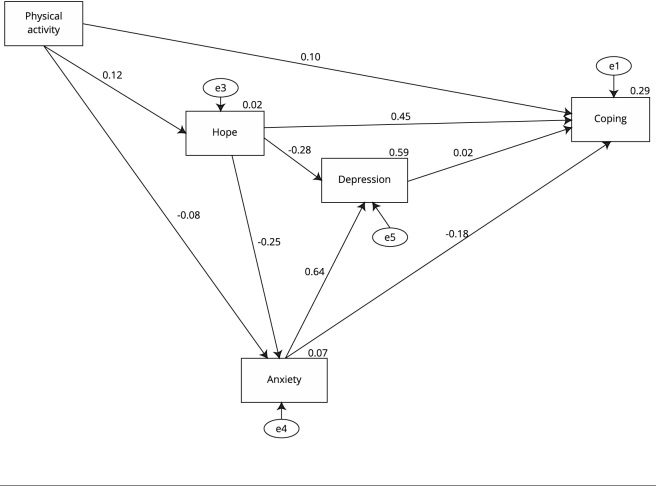
Structural-equation modeling diagram of physical activity, hope, depression, anxiety, and coping levels among adults of Turkey during COVID-19 lockdown.

Table 2 presents structural equation modeling results, which indicate direct influence of physical activity on coping (β = 0.10, p < 0.01), hope (β = 0.12, p < 0.01) and anxiety (β = -0.08, p < 0.01). As the central factor of this study, hope directly and positively influenced coping (β = 0.45, p < 0.01) and directly and negatively influenced anxiety (β = -0.25, p < 0.01) and depression (β = -0.28, p < 0.01). Anxiety also affected depression (β = 0.64, p < 0.01) and coping directly (β = -0.18; p < 0.01). Depression did not significantly impact coping. Physical activity indirectly affected coping via hope, anxiety, and depression (standardized total effect - STe = 0.173). Hope also indirectly affected coping by impacting depression and anxiety (STe = 0.486). Generally, the structural model of this study explained 29% of the variation in coping (Figure 1). Model fit indexes presented good model-data fit for the proposed framework (Table 3).

**Table 2:** Relative contributions of the predictor variables to prediction of coping.

Predictor	Dependent variable	β	p-value	t test	SE	STe
Physical activity	Coping	0.10	< 0.00	4.37	0.070	0.173
Physical activity	Hope	0.12	< 0.00	4.62	0.014	0.123
Physical activity	Anxiety	-0.08	0.002	-3.14	0.030	-0.112
Physical activity	Depression	-	-	-	-	-0.106
Hope	Coping	0.45	< 0.00	17.66	0.140	0.486
Hope	Anxiety	-0.25	< 0.00	-9.54	0.560	-0.248
Hope	Depression	-0.28	< 0.00	-15.85	0.038	-0.441
Anxiety	Depression	0.64	< 0.00	36.05	0.018	0.642
Depression	Coping	-0.02 *	0.58	0.55	0.092	0.019
Anxiety	Coping	-0.18	< 0.00	-5.63	0.086	-0.170

SE: standard error; STe: standardized total effect.

Note: STe normalizes the total effect by accounting the ratio between standard deviations of the variables (x) and the target node (y).

* Not significant; R^2^Coping = 0.29; R^2^Anxiety = 0.07; R^2^Depression = 0.59; R^2^Hope = 0.02.

**Table 3:** Model fit indices.

	χ^2^ (df)	IFI	NFI	GFI	CFI	RMR	RMSEA
Model	1.211 (1)	1.00 *	0.99 *	1.00 *	1.00 *	0.004 **	0.012 ***

CFI: *comparative fit index;* GFI: goodness-of-fit index; IFI: incremental fit index; NFI: normed fit index; RMR: root mean square; RMSEA: root mean square error of approximation.

* IFI, NFI, GFI, & CFI > 0.90;

** RMR < 0.05;

*** RMSEA close to 0.05.

Table 4 presents the independent variables (e.g., physical activity and gender) effects on the four dependent variables. Considering direct effect on the analysis, in Model 1, the effect of physical activity on hope was positive (β = 0.54, p < 0.01) while gender had no effect (p > 0.01); in Model 2, the effect of physical activity on coping was positive (β = 0.41, p < 0.01) and gender was negative (β = -0.22, p < 0.01); In Model 3, the effect of physical activity on anxiety was negative (β = -0.06, p < 0.01) and gender was positive (β = 0.27, p < 0.01); in Model 4, the effect of physical activity on depression was negative (β = -0.08, p < 0.01) and gender was positive (β = 0.22, p < 0.01). When gender was analyzed as mediator, gender had no effect on the relation between physical activity and dependent variables in all four models (p > 0.01).

**Table 4:** Gender mediating effect on physical activity and hope, coping, anxiety, and depression relation.

Paramenter	Model 1: hope			Model 2: coping			Model 3: anxiety			Model 4: depression		
	β	SE	p-value	β	SE	p-value	β	SE	p-value	β	SE	p-value
Constant	3.42	0.122	0.00	15.38	0.068	0.00	2.47	0.025	0.00	2.10	0.025	0.00
Physical activity	0.54	0.122	0.00	0.41	0.068	0.00	-0.06	0.025	0.00	-0.08	0.025	0.00
Gender	-0.12	0.122	0.32	-0.22	0.068	0.00	0.27	0.025	0.00	0.22	0.025	0.00
Int_1 (mediator)	0.00	0.122	0.53	-0.00	0.068	0.99	-0.00	0.025	0.75	0.00	0.025	0.71
F	7.543			17.959			47.552			33.255		
p-value	0.00			0.00			0.00			0.00		
R^2^	0.016			0.037			0.092			0.066		

F: variance; Int_1: mediator; SE: standard error.

## Discussion

This study examined adults physical activity, hope, depression, anxiety, and coping status by gender during the COVID-19 pandemic, the effects of physical activity, hope, depression, and anxiety on coping with the pandemic, and the effect of gender on the relation between physical activity and hope, coping, depression, anxiety.

This study shows the existence of significant differences in physical activity levels, depression, anxiety, and coping scores by gender, but no significant difference were found by gender for hope scores. Men had higher levels of physical activity and higher coping score than women, whereas women had higher anxiety and depression scores than men. Women having lower physical activity levels than men and a more sensitive structure may have increased their anxiety and depression levels to higher scores than men in this process. Maugeri et al. ^27^ reported that physical activity levels decreased in both men and women during the pandemic compared to before the pandemic. In particular, women physical activity levels decreased more during the pandemic ^27^ due to men preferring to participate in outdoor team sports for social and competitive reasons, while women prefer indoor exercises (aerobics, pilates, dance, yoga, etc.) ^28^. The decreased physical activity levels due to the pandemic conditions pose a health threat, both physical and mental ^27^. Etheridge & Spantig ^29^ examined the gender gap in the U.K. population’s mental well-being during the COVID-19 pandemic and discovered that women experienced greater decline in mental well-being than men during the pandemic. Similar findings are reported by Bäuerle et al. ^30^, Hyland et al. ^31^, and Salari et al. ^3^. Similarly, the reported reason for higher prevalence of anxiety and depression among women during the pandemic ^3^ ^,^ ^32^ ^,^ ^33^ ^,^ ^34^ is that they struggle to cope with limited physical activity opportunities and increased responsibilities, due totheir emotional and fragile structure ^35^. In addition, no significant gender-based difference was found regarding hope. Some researchers have already observed that women and men disregard hope as a valuable coping mechanism ^36^ ^,^ ^37^. Mansur & Doğuç ^38^ similarlly reported that no gender difference was found in hope levels of university studentsduring the COVID-19 pandemic.

Our study reports that physical activity, hope, depression, and anxiety were significantly associated with coping during COVID-19 period. These variables can directly or indirectly impact one’s ability to deal with COVID-19 the pandemic. Physical activity and hope positively affected coping with COVID-19, whereas anxiety negatively impacted the coping. Hope negatively influenced anxiety and depression, and depression negatively affected coping by anxiety. Our findings support the idea that greater physical activity and hope levels have a positive effect on coping with COVID-19. Physical activity is considered vital for mental fitness maintenance and has been recognized as an effective coping strategy to deal with stress. Studies show that physical activity produces endorphins - a chemical that acts as natural painkiller in the brain - and also improves sleep, which reduces stress ^39^. Evidence suggests that regular participation in physical activity decreases overall levels of tension and makes one feel energized and healthy. Also, physical activity may be therapeutic for people with severe mental illness who generally have low physical activity and experience numerous lifestyle-related medical complications ^40^. Similarly, the study conducted by Mledenova ^39^ suggests that regular participation in physical activity decreases overall levels of anxiety and depression as a reliable coping strategy to overcome social isolation and, as a whole, the negative aspects of lockdown. All these studies are consistent with the relationship between physical activity, anxiety and depression also revealed in our outcomes. Moreover, many studies reveal hope as an important resource in coping with stress, uncertainty ^41^ and psychological adaptation ^42^ ^,^ ^43^. Khalaf et al. ^44^ emphasized that coping is important to handle the consequences of stressful events, such as anxiety, depression and other psychological distress. Additionally, Gallager et al. ^12^ observed that as hope scores increased, depression and anxiety scores decreased. Amirav et al. ^45^ compared the hope levels of adults before and after the pandemic and found that they were inversely proportional to depression and loneliness levels. Sinclair & Wallston ^24^ found that coping and hope levels were associated with reduced anxiety and depression symptoms. Consistent with our study results, Faulkner et al. ^9^ emphasized that depression and anxiety negatively affect physical activity, hope and coping, and the positive effect of physical activity on mental health. Therefore, physical activity is an important coping tool to maintain or improve physical and mental health during the pandemic. In light of this evidence, we can state that increased physical activity levels reduce depression and anxiety, improve levels of hope and ultimately lead to better health during COVID-19 period.

Our results proved that physical activity directly affects coping, hope, anxiety, and depression. Accordingly, it is understood that physical activity increases coping and hope and decreases anxiety and depression. Also, it was found that gender has no effect on the relation between physical activity and hope, coping, anxiety, and depression, although gender was associated with decreased coping and increased depression and anxiety. Individuals may have fear-oriented thoughts in the face of negative events. Such thoughts may trigger feelings of anxiety, depression and the inability to cope with such reality. However, when factors such as physical activity and hope are considered, individuals’ attitudes towards negative events may change. León-Zarceño et al. ^46^ report that psychologic health (psychological well-being, coping, emotions, and perception of daily difficulties) - considering gender, and perceived changes in life - support gender-based difference especially for perceived emotions, difficulties for certain routines, psychological health, and coping, although no significant gender-based differences were found regarding physical activity levels. A comprehensive meta-analysis conducted by Netz & Wu ^47^ examined data from 36 studies linking physical activity and adults well-being and confirms the findings for the positive physical activity effects on general psychological health. Additionally, Clemente-Suárez et al. ^48^ found that physical activity and exercise reduces COVID-19-related psychological symptoms, increases hope, and strengthens one’s self-confidence ^49^. These findings support physical activity as a coping tool during the COVID-19 pandemic, as it significantly contributes to both physical and mental health ^9^. When evaluated from this perspective, our study results show the importance of physical activity and exercise in maintaining hope to better cope with COVID-19.

### Limitations and future directions

This study has several limitations. Firstly, because this study used a convenience sample, so the participants may not truely represent Turkish population and the results cannot be generalized. Thus, our results may have suffered from selection bias. Secondly, our results should be cautiously interpreted, because the study design is cross-sectional; causality cannot be inferred from our results. Lastly, data collection included a retrospective self-report method, and recall bias may have affected individual results. Future research evaluating physical activity behaviors and psychological factors related to the post-pandemic normalization period will better help in understanding the role of physical activity in coping with mental health challeges.

## Conclusion

This study results support that adults physical activity during COVID-19 lockdowns were positively correlated with increased hope and coping levels. On the other hand, lack of physical activity was correlated with increased levels of anxiety and depression. Moreover, higher physical activity and hope levels directly and positively affected coping with the pandemic, while hope was the best coping predictive variable. In addition, gender has no effect on the relation between physical activity and hope, coping, anxiety, and depression. These results demonstrate the importance of physcical activity and hope to cope with COVID-19.

### Recommendations

This study findings showed that physical activity and hope directly and positively influenced coping with the COVID-19 pandemic. In this context, building an environment where adults can engage in regular physical activity and exercise to facilitate coping with COVID-19 is important, as it improves focus and motivation, while having fun, a natural energy boost, and alleviating tension, stress, mental fatigue, anger and frustration. Additionally, preventive physical activity and exercise interventions should be adapted to specific sociodemographic factors such as gender and should consider lifestyle impact on emotional well-being. Moreover, identifying other factors that cause hopelessness, anxiety and depression in adults and offering institutional solutions is also important.
